# Fluopyram Induces Multilevel Toxicity in Zebrafish: Insights from Developmental Impairment, Oxidative Stress, and Metabolic Disruption

**DOI:** 10.3390/jox16020069

**Published:** 2026-04-20

**Authors:** Ningbo Wang, Yingying Zhong

**Affiliations:** 1 Ningbo Customs Technology Center, Ningbo 315012, China; 2 School of Food Science and Biotechnology, Zhejiang Gongshang University, Hangzhou 310018, China

**Keywords:** fluopyram, zebrafish, developmental toxicity, metabolic disorder, hepatotoxicity

## Abstract

Fluopyram (FO), a widely used succinate dehydrogenase inhibitor (SDHI) fungicide, poses a potential risk to aquatic ecosystems due to its mitochondrial toxicity in non-target organisms. This study investigated its toxic effects on zebrafish (*Danio rerio*). Embryos (*n* = 30 per concentration) were exposed to FO (0, 0.375, 0.75, 1.5 mg/L) for 96 h, resulting in concentration-dependent developmental toxicity, including increased malformations, reduced heart rate, and inhibited swimming behavior. Adult zebrafish were chronically exposed to lower concentrations (0, 0.01, 0.1, 1.0 mg/L; *n* = 20 per concentration per replicate) for 28 days. Biochemical analyses across both life stages revealed that FO significantly inhibited succinate dehydrogenase (SDH) activity and mitochondrial complex II, reduced ATP levels, and induced oxidative stress. Integrated transcriptomic and metabolomic analyses showed that FO profoundly perturbed specific metabolic pathways, primarily glutathione metabolism, cytochrome P450-mediated detoxification, and core nutrient metabolism pathways involving carbohydrates, lipids, and amino acids. In adults, chronic exposure induced significant hepatotoxicity, evidenced by histopathological damage, altered liver enzyme activities (GPT/GOT), and activation of autophagy and PPAR/FoxO signaling pathways. Our results demonstrate that FO induces multifaceted toxicity in zebrafish, from developmental defects to hepatic metabolic dysfunction, primarily driven by mitochondrial impairment and oxidative stress. This study provides crucial mechanistic hazard data and insights for the ecological risk assessment of SDHI fungicides.

## 1. Introduction

The widespread use of pesticides poses a potential threat to water ecosystems and biological health while safeguarding crop yield and quality [1,2]. Succinate dehydrogenase inhibitor (SDHI) fungicides are an important part of modern pesticides that inhibit succinate dehydrogenase in the mitochondria of fungal pathogens, blocking their energy metabolism with a fungicidal activity [3]. However, as a key enzyme in the tricarboxylic acid cycle and electron transport chain, SDH is widely found in most eukaryotic organisms. Thus, while efficiently killing fungi, SDHI pesticides are also toxic to non-target organisms, including animals and plants, especially aquatic organisms. [4,5,6].

Fluopyram (FO), one of the SDHI fungicides, has been widely used for the control of fruit and vegetable diseases and nematodes since its promotion in 2013 due to its broad-spectrum and highly effective properties [7,8]. Monitoring data have shown that FO can enter water bodies through surface runoff, and its residues have been detected in aquatic environments in several regions around the globe at concentrations of up to 1.0 µg/L, suggesting a potential aquatic ecological risk [9,10]. Monitoring of rivers and springs in northern Spain identified FO as the most frequently detected pesticide; its stable structure confers persistence in both water and soil, with half-lives reaching ≥207 days [10]. Zebrafish has become a classic aquatic model for assessing the toxicity and metabolic behavior of environmental pollutants due to its genome being highly homologous with humans, as well as its embryonic transparency and rapid development [11]. Because the target of FO’s action, SDH, is highly conserved among all eukaryotes, previous studies have found that a variety of SDHI-based pesticides are developmentally, cardiovascularly, and neurobehaviorally toxic to aquatic organisms, such as zebrafish [12,13,14]. SDHI pesticides, such as Boscalid and Isopyrazam, cause cardiac toxicity and neurobehavioral toxicity in zebrafish, can cause heart rate abnormalities and oxidative stress, and can impair mitochondrial function [15,16].

Previous studies have demonstrated that FO induces adverse effects in a variety of non-target organisms. In *Caenorhabditis elegans*, FO exposure can induce oxidative stress, intestinal damage, and cell apoptosis [17,18]. In honey bees (*Apis mellifera*), FO inhibits the critical energy metabolism critical enzyme SDH, thereby posing a threat to colony health [19]. In mammals, FO activates nuclear receptors (CAR/PXR) in human hepatocytes, inducing the expression of cytochrome P450 enzymes (CYP3A4) [20]. Chronic exposure in rats and mice has been shown to mediate the formation of liver and thyroid tumors [21,22], suggesting a potential human health risk. In fish, studies have confirmed that FO undergoes biotransformation and bioaccumulation in zebrafish [23] and can affect bone development in combination with other pesticides [24]. Consequently, the potential hazards of FO to aquatic organisms and human health have become a focus in both ecotoxicology and environmental toxicology. There is an urgent need for research on the toxic mechanisms of FO in zebrafish to provide theoretical guidance for risk assessment. However, there is still a lack of studies on the toxic effects of FO on zebrafish systems and its mechanisms.

This study aimed to systematically investigate the multilevel toxicity and elucidate the underlying mechanisms of FO in zebrafish. We aimed to evaluate FO toxicity and explore potential mechanisms by measuring key enzymes, gene expression, and metabolites, providing data relevant to environmental safety assessment.

## 2. Materials and Methods

### 2.1. Chemicals and Reagents

Fluopyram (>99% purity, CAS: 658066-35-4) was purchased from Dr. Ehrenstorfer GmbH (Augsburg, Germany) and a stock solution of 20,000 mg/L fluopyram was prepared with analytical grade acetone stored at 4 °C. All chemicals used in this study were of analytical grade. Reconstituted water containing 2 mmol/L Ca^2+^, 0.5 mmol/L Mg^2+^, 0.75 mmol/L Na^+^ and 0.074 mmol/L K^+^ was prepared in the laboratory.

### 2.2. Zebrafish Culture and Exposure Experiments

Adult zebrafish were obtained from the Institute of Aquatic Biology, Chinese Academy of Sciences (Wuhan, China), and cultured in 10 L glass tanks filled with 8 L of dechlorinated tap water, with a light period of 14:10 h, a temperature of 28 ± 0.5 °C, and a pH value of 7.2–7.8. The water quality was maintained within the following ranges: pH 7.2–7.8, dissolved oxygen 6–8 mg/L, conductivity 30–70 μS/cm, and ammonia/nitrite levels below 0.1 mg/L. *Artemia* nauplii were administered twice daily (at 9:00 and 17:00). No unusual mortality of zebrafish occurred during this period. Gender is not distinguished in hepatotoxicity studies using adult zebrafish. All animal procedures were conducted in accordance with the guidelines approved by the Experimental Ethics Committee of Zhejiang Gongshang University (protocol code 2025-1-320-31).

Fertilized zebrafish embryos within 90 min post fertilization were selected and randomly assigned, and the experiment was set up with control and exposure groups of 30 zebrafish embryos each. Three parallels were set up for each exposure concentration. They were placed in a constant temperature light incubator for incubation (28 ± 1 °C), and the exposure test solution was changed daily. The daily mortality rate of the embryos, 24 h spontaneous tail movement, and 48 h heart rate were recorded, and micrographs of embryonic malformations were taken. We replaced the test solution daily, recorded mortality rates, and removed dead eggs and post-hatch egg membranes. We calculated the 96h-LC_50_ using the Probit analysis method based on mortality data.

Five-month-old zebrafish were randomly assigned to be exposed to FO at concentrations of 0, 0.01, 0.1, and 1.0 mg/L for 28 days; acetone (0.0001% *v*/*v*) served as the solvent control group. In this study, the 96h-LC_10_ concentration of an adult fish (1.0 mg/L) was set as the maximum exposure concentration. There were three replicate pools of 20 fish per concentration. The 96h-LC_10_ and 96h-LC_50_ values for zebrafish embryos and adults are displayed in Table S1. Details of the zebrafish embryo acute toxicity assay are provided in Supplementary Text S1. During semi-static exposure, solutions were replaced daily with freshwater containing the corresponding concentration of FO. To further clarify the stability of FO in exposed water, the exposure solutions were determined by liquid chromatography and the data are presented in Table S2. Liver tissues were collected from adult fish after exposure and stored in a −80 °C refrigerator for biochemical and histological assays.

### 2.3. Behavioral Analysis

Light stimulation was used to assess the effect of FO on the swimming behavior of embryos. Zebrafish embryos were developed to 120 hpf. There were 24 per treatment group, and three experimental replicates were set up. We placed each zebrafish in a dedicated behavioral analysis chamber and inserted it into the ZebraBox (ViewPoint; ZebraLab 3.3, Lyon, France) behavioral analysis system. The light–dark sequence was set as dark, light, dark, light, dark, with each light cycle lasting 10 min. The total duration was set to 50 min, and zebrafish behavioral data were collected every minute. Video-Track motion trajectory analysis software (version 3.3) was used to detect and analyze the swimming speed and distance data of zebrafish embryos.

### 2.4. Analysis of Biochemical and Antioxidant Indices

Zebrafish embryo or liver samples were homogenized on ice in pre-cooled phosphate-buffered saline (PBS, 0.01 M, pH 7.2–7.4) using a mechanical homogenizer (Sangong Biotech, Shanghai, China). The homogenate was then centrifuged at 4 °C (8000× *g* for 10 min) to collect the supernatant, and assayed for superoxide dismutase (SOD, A001-3-2), catalase (CAT, A007-1-1), glutathione (GSH, A006-2-1), succinate dehydrogenase (SDH, A022-1-1), mitochondrial respiratory complex II (respiratory chain II, A022-1-1), adenosine triphosphate (ATP, A095-1-1), glucose-6-phosphate dehydrogenase (G6PDH, A062-1-1) activity, alanine aminotransferase (ALT(GPT), C009-2-1), aspartate aminotransferase (AST(GOT), C010-2-1), enzyme activities, or content indicators according to the kit instructions provided by the manufacturer [25]. The detailed detection principles and key operational steps for each assay are summarized in Supplementary Text S2. The total protein concentration of each supernatant was quantified using the bicinchoninic acid (BCA) protein assay kit to normalize the enzyme activity or metabolite content data (expressed per mg of protein). All assays were performed in triplicate.

### 2.5. HE-Stained Sections of Zebrafish Tissues

Zebrafish were dissected after 28 d of exposure, and liver samples were collected and fixed in 4% paraformaldehyde at 4 °C for 24 h. Liver samples were randomly selected from five fish per group, with three non-consecutive sections examined per sample. Gradient dehydration was carried out with alcohol, followed by transparency with xylene, and subsequent paraffin embedding. After embedding, they were cut into consecutive sections with a thickness of 5 μm, the slices and patches were sectioned and stained with hematoxylin and eosin for dewaxing, dehydrated and transparent again with alcohol and xylene, and finally sealed with resin for upright optical photography microscope (Nikon, Tokyo, Japan) observation and photography. Liver histopathological changes were semi-quantitatively assessed using the Bernet scale to quantify the severity of liver injury [26]. The corresponding scores and organ indices are summarized in Table S3.

### 2.6. Transcriptome Analysis

Zebrafish embryos and adult livers from the FO and control groups were dissected and frozen in liquid nitrogen, and total RNA was analyzed qualitatively and quantitatively using a Nano Drop and Agilent 2100 Bioanalyzer (Thermo Fisher, Waltham, MA, USA). Subsequently, transcriptome sequencing was performed using the BGIseq500 platform (BGI-Shenzhen, China). Raw sequencing data were filtered and analyzed according to the database established by BGI. Differentially expressed genes (DEGs) were identified using DESeq2. GO and KEGG enrichment analyses were performed using a hypergeometric test (phyper). Raw sequencing data were stored in the Genome Sequence Archive (Accession number PRJCA054276).

### 2.7. Metabolomics Assay

An appropriate amount of zebrafish liver tissue was taken, and the metabolites were extracted by adding extraction reagent (methanol/acetonitrile/water (2:2:1, *v*/*v*/*v*)). Chromatographic separation was performed on a Waters ACQUITY UPLC BEH C_18_ column (1.7 μm, 2.1 mm × 100 mm, Waters, Milford, MA, USA) equipped with a heated electrospray ionization source and a Q-Exactive mass spectrometer (Thermo Fisher Scientific, Waltham, MA, USA). Compound Discoverer 3.1 (Thermo Fisher Scientific, Waltham, MA, USA) software was used for LC-MS/MS data processing, and data preprocessing, statistical analysis, metabolite classification annotation, and functional annotation were performed using the metabolomics R package meta X (version 1.4.2).

### 2.8. Statistical Analysis

All experimental data are presented as the mean ± standard deviation. Statistical comparisons among multiple exposure groups for biochemical indices were performed using one-way analysis of variance (ANOVA) followed by Tukey’s post hoc test for pairwise comparisons. Data normality and homoscedasticity were verified. Where ANOVA assumptions were not met, the Kruskal–Wallis test with Dunn‘s post hoc test was employed. To control the false discovery rate (FDR) across multiple endpoints, *p*-values were adjusted within predefined biological endpoint families using the Benjamini–Hochberg procedure. Specifically, the biochemical assays for embryos and those for adult livers were treated as separate families. Within each family, the adjustment accounted for all pairwise comparisons among the four exposure groups (i.e., 6 comparisons per measured endpoint). A significance threshold of adjusted *p* < 0.05 was applied. For transcriptomic and metabolomic analyses, differentially expressed genes and metabolites were identified with thresholds of |log_2_(fold change)| ≥ 1 and an FDR-adjusted *p*-value (Q-value) ≤ 0.05 using established bioinformatics pipelines (DESeq2 and metaX), which inherently account for multiple testing. A *p*-value (or Q-value) < 0.05 was considered statistically significant. In figures, significance is denoted as follows: * *p* < 0.05, ** *p* < 0.01, *** *p* < 0.001. All analyses were conducted using GraphPad Prism 8 and the respective omics software.

## 3. Results

### 3.1. Developmental Toxicity of Zebrafish Embryos

**Figure 1:**
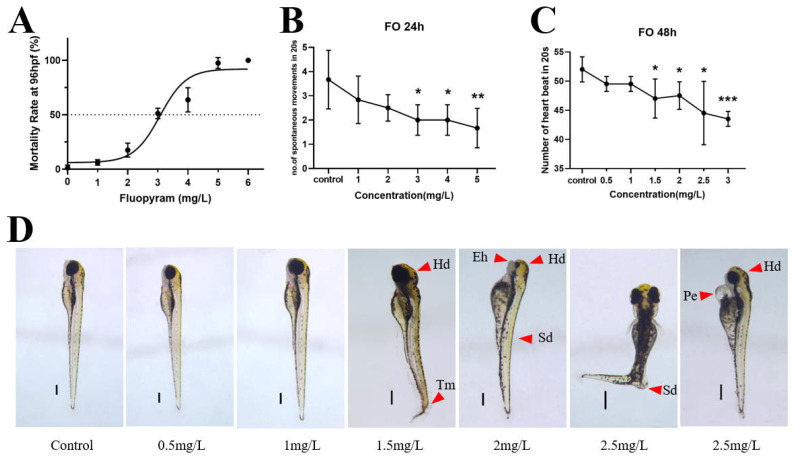
96 h lethal curve of zebrafish embryo (**A**), 24 h spontaneous tail movement (**B**), 48 h heart rate (**C**), and 72 h aberration micrograph of zebrafish embryo (**D**). Significance of differences is labeled as * *p* < 0.05; ** *p* < 0.01; *** *p* < 0.001. (The scale bar is 50 μm.)

The embryonic lethality curve was plotted based on acute exposure of zebrafish (Figure 1A). The 96h-LC_50_ value of FO for zebrafish embryos was 3.01 mg/L, with a 95% Confidence limit of 2.67–3.35 mg/L (Table S1). After 24 h of exposure, FO exposure above 3 mg/L significantly decreased the spontaneous tail movements of zebrafish embryos, and FO exposure above 1.5 mg/L significantly decreased their 48 h heart rate. As shown in Figure 1D, exposure to FO at 1.5 mg/L or above induced abnormal head development (Hd), hypoplasia of the eye point (Eh), abnormal curvature of the spine (Sd), folded tail (Tm), and pericardial edema (Pe) in zebrafish embryos at 72 h. These results show that FO toxicity causes developmental abnormalities in zebrafish embryos.

**Figure 2:**
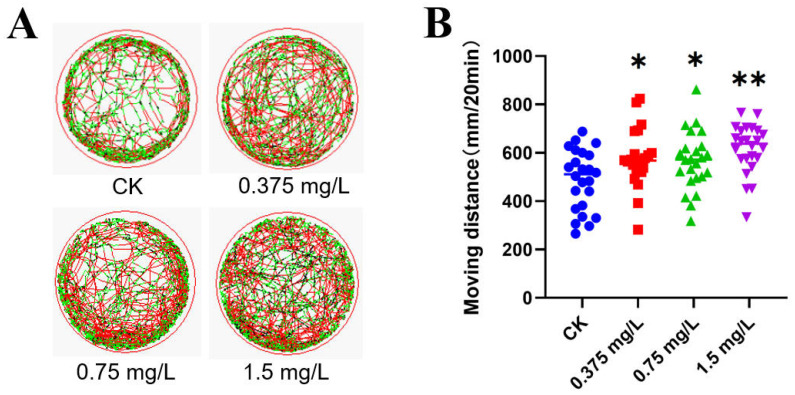
Results of 120 h embryonic swimming assay of zebrafish. Swimming line diagram (**A**) with trajectories colored according to the light–dark cycle (green: light; red: dark), swimming distance (**B**). Significance of differences is labeled as * *p* < 0.05; ** *p* < 0.01.

**Figure 3:**
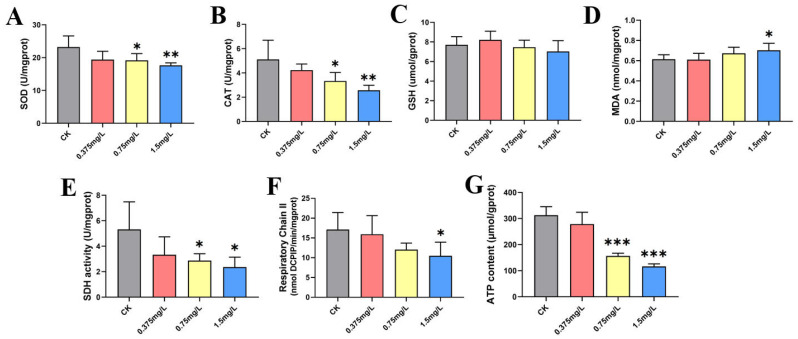
Oxidative stress and mitochondrial biochemical indices in zebrafish embryos. The activities of (**A**) superoxide dismutase (SOD) and (**B**) catalase (CAT) as well as the contents of (**C**) reduced glutathione (GSH) and (**D**) malondialdehyde (MDA) were measured. The activities of (**E**) succinate dehydrogenase (SDH) and (**F**) mitochondrial respiratory chain complex II, along with (**G**) adenosine triphosphate (ATP) content, were also determined. Data are presented as mean ± standard deviation (SD) (*n* = 30). Significance of differences is labeled as * *p* < 0.05; ** *p* < 0.01; *** *p* < 0.001.

Behavioral trajectory analysis of gradient exposure experiments based on the 96h-LC_50_ of half of the zebrafish embryos shows that FO exposure significantly increased their swimming distance (120h) (see Figure 2). As shown in Figure 3, the SOD activity of zebrafish embryos gradually decreased with increasing FO exposure concentration, and the activity of the antioxidant enzyme CAT was also gradually reduced. The GSH content did not change significantly after FO exposure, whereas the MDA content was significantly increased in the 1.5 mg/L exposure group. FO induced a significant oxidative stress response in zebrafish embryos. The activity of SDH, the target site of succinate dehydrogenase of FO, was significantly inhibited by FO. The activity of SDH, the target site of succinate dehydrogenase, was significantly inhibited by FO, and the activity of respiratory chain complex II was also significantly inhibited in the 1.5 mg/L exposure group, while the ATP content of zebrafish embryos was significantly reduced in the 0.75 mg/L and 1.5 mg/L exposure groups. FO significantly inhibited SDH activity and mitochondrial energy supply of zebrafish embryos.

### 3.2. Transcriptional Analysis of Zebrafish Embryos

**Figure 4:**
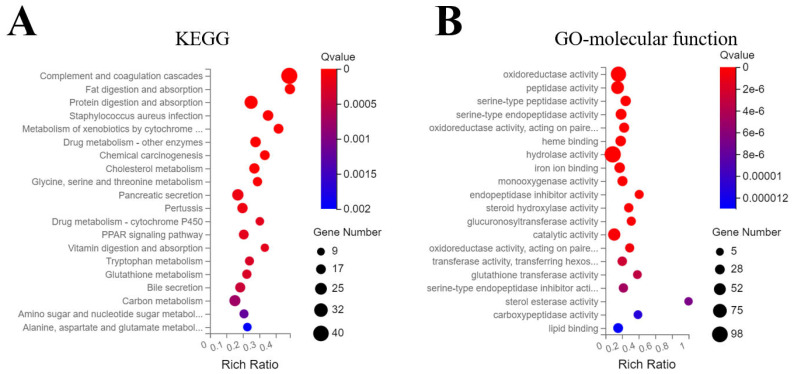
Transcriptomic analysis of zebrafish embryos exposed to FO. Embryos were exposed to 0.75 mg/L FO or solvent control for 96 h. (**A**) Significantly enriched Kyoto Encyclopedia of Genes and Genomes (KEGG) pathways among differentially expressed genes. (**B**) Significantly enriched molecular function terms from Gene Ontology (GO) annotation.

Transcriptomic analysis identified 25,404 expressed transcripts. Using |log2FC| ≥ 1 and Q ≤ 0.01, 989 genes were differentially expressed (32 down-regulated, 957 up-regulated). As shown in Figure 4A, KEGG enrichment analysis revealed that FO mainly perturbed two signaling pathways; firstly, toxicant metabolism, glutathione and P450 detoxification metabolism related pathways were activated, and secondly, nutrient metabolism pathways affecting sugar, fat, amino acid, and protein metabolism. Consistently, GO molecular function enrichment indicated the activation of redox- and glutathione-related enzymes, as well as those enzymes involved in carbohydrate and lipid metabolism.

### 3.3. Hepatotoxicity of Adult Zebrafish

**Figure 5:**
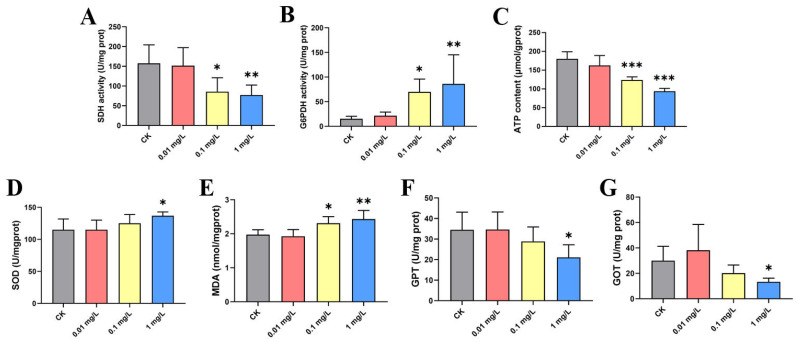
The effects of chronic FO exposure on biochemical parameters in adult zebrafish. Adult zebrafish were exposed to FO at concentrations of 0, 0.01, 0.1, and 1.0 mg/L for 28 days. The activities of (**A**) succinate dehydrogenase (SDH) and (**B**) glucose-6-phosphate dehydrogenase (G6PDH), (**C**) adenosine triphosphate (ATP) content, (**D**) superoxide dismutase (SOD) activity, (**E**) malondialdehyde (MDA) content, and the activities of (**F**) alanine aminotransferase (GPT) and (**G**) aspartate aminotransferase (GOT) were measured in liver tissue. Significance of differences is labeled as * *p* < 0.05; ** *p* < 0.01; *** *p* < 0.001.

**Figure 6:**
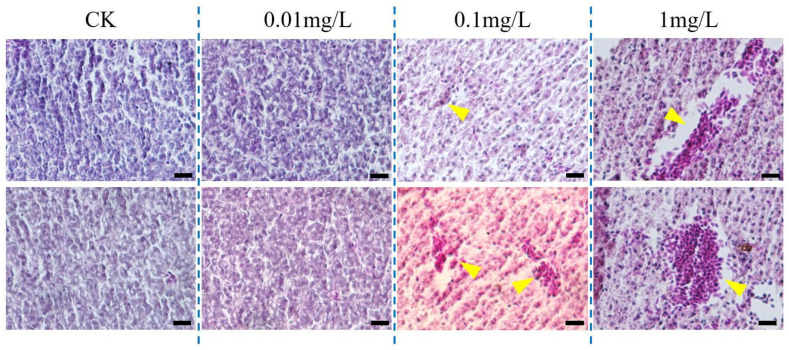
Histopathological alterations in the liver of adult zebrafish after chronic exposure to FO. Representative hematoxylin and eosin (H&E)-stained sections of liver tissue from zebrafish exposed to CK (solvent control), 0.01, 0.1, and 1.0 mg/L FO for 28 days. Yellow arrowheads indicate nuclear pyknosis and hepatocellular necrosis. The scale bar is 50 μm.

The 96h-LC_50_ of FO on adult zebrafish was 9.962 mg/L (Table S1). Three FO concentrations, 0.01, 0.1, and 1.0 mg/L, were selected for 28-day exposure of adult zebrafish. As shown in Figure 5, FO exposure significantly decreased SDH activity and ATP content, significantly increased the activity of the rate-limiting enzyme of the pentose phosphate pathway, G6PDH, promoted the activity of SOD, and significantly increased the content of MDA, resulting in oxidative stress damage. As shown in Figure 5E,F, chronic exposure also induced a significant decrease in the activities of GPT and GOT measured in liver homogenates. HE-stained sections (Figure 6) revealed that chronic FO exposure induced dose-dependent histopathological damage in zebrafish liver. While the 0.01 mg/L group showed no significant alterations compared to the control, the 0.1 mg/L group exhibited mild lesions, including focal necrosis and vacuolar degeneration. The 1.0 mg/L group displayed severe and diffuse damage characterized by extensive necrosis, vacuolization, nuclear pyknosis, and inflammatory infiltration. Semi-quantitative analysis using the Bernet scale (Table S3) demonstrated a significant increase in the liver histopathological index in both the 0.1 mg/L and 1.0 mg/L exposure groups compared to the control (*p* < 0.01 and *p* < 0.001, respectively), providing quantitative evidence that corroborates the biochemical markers of hepatotoxicity.

### 3.4. Transcriptome and Metabolome Analysis of Zebrafish Adults

**Figure 7:**
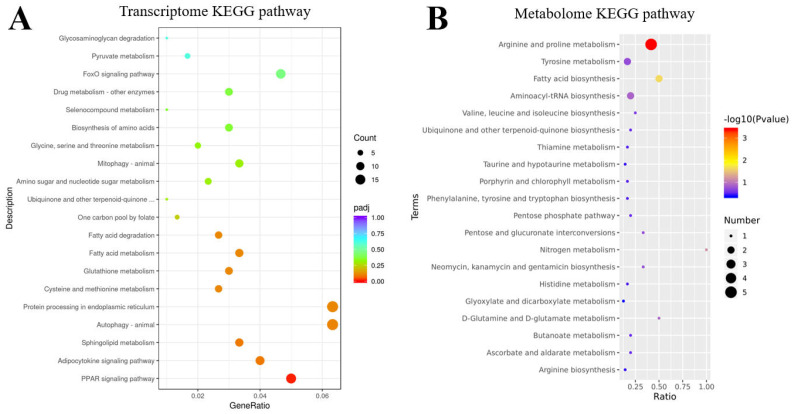
Integrated omics analysis of the liver from adult zebrafish chronically exposed to FO. Adult zebrafish were exposed to 0.1 mg/L FO or solvent control for 28 days. Significantly enriched Kyoto Encyclopedia of Genes and Genomes (KEGG) pathways from the (**A**) transcriptomic and (**B**) metabolomic analyses are shown.

There were 986 significantly different genes in the transcriptome of zebrafish adult liver in the control and 0.1 mg/L FO-exposed groups, of which 381 were up-regulated and 605 were down-regulated. As shown in Figure 7A, KEGG enrichment analysis of the transcriptome showed that FO mainly affected nutrient metabolic processes, including pyruvate, fatty acid, amino acid, and nucleotide metabolism and glycan degradation. Similarly to the embryonic transcriptome, detoxification and glutathione metabolism pathways were affected, with the adult liver transcriptome being particularly enriched for the cellular autophagy, Foxo, PPAR, and cytokine cell signaling pathways. GSEA enrichment analysis that did not depend on significant differences showed that cellular autophagy and mitochondrial phagocytosis are associated with adipocytosis and functional sugar metabolism (Table S4). This suggests that FO induces not only detoxification and nutrient metabolism processes in adult fish liver, but also cellular autophagy and a series of transcription factor regulations.

## 4. Discussion

The toxicity of FO to zebrafish embryos was lower compared to the lethal toxicity of SDHI fungicides, with an LD_50_ of 0.066 mg/L for benzovindiflupyr and 1.1 mg/L for bixafen [27,28]. However, in common with other SDHI fungicides, FO exhibits strong inhibition of SDH and inhibition of ATP production and mitochondrial function, which may in turn contribute to oxidative stress in zebrafish embryos [29]. FO causes a decrease in heart rate and a variety of developmental malformations in developing zebrafish embryos. Behaviorally, FO leads to an increase in swimming. SDHI-based fungicides have been found to alter behavioral aspects such as locomotion and feeding in zebrafish embryos through mitochondrial inhibitory effects and, consequently, behavioral changes [7,30]. The KEGG and GO enrichment analysis of the transcriptome suggested that FO inhibition of SDH activity, leading to decreased ATP content and an energy supply shortage, may activate detoxification pathways involving glutathione and P450 systems, while the increased SOD and CAT activities might enhance antioxidant function to counteract FO-induced stress. It is reported that zebrafish cytochrome P450 enzymes metabolize FO via oxidation and hydroxylation, leading to its detoxification [23]. On the other hand, the oxidative phosphorylation pathway reduces the efficiency of ATP production because of the inhibition of SDH activity, so nutrients additionally increase the energy supply to the cell through processes such as the pentose phosphate pathway, lipid degradation metabolism, and amino acid conversion [31].

In chronic exposure in adult fish, FO not only inhibits mitochondrial function and generates oxidative stress, but also causes significant toxic effects of liver damage. As the liver is the main organ for toxic metabolism and nutrient transformation in zebrafish, FO-induced mitochondrial inhibition and nutrient metabolism disruption caused organic liver lesions during long-term exposure [32]. SDHI pesticides such as bixafen and thifluzamide and boscalid were also found to cause hepatotoxic injury accompanied by the formation of nutrient metabolism including lipids and sugars [33,34,35]. Decreased GPT and GOT activity observed in liver tissue, contrary to the typical upward trend in serum, indicates the late stage of hepatotoxicity, which may be attributed to persistent oxidative stress leading to functional hepatocyte necrosis, reducing total enzyme content [36]. Combined with histopathological evidence, this confirms FO-induced liver injury following chronic exposure.

Combined with histopathological evidence, these findings support the conclusion of FO-induced liver injury following chronic exposure. Compared to the research by Zhou et al. (2025) [23], which detailed the bioconcentration and biotransformation of FO in adult zebrafish at sublethal concentrations (up to 1.80 mg/L for 14 days), our work provides a complementary perspective by systematically investigating its toxic effects across different life stages. We not only examined acute developmental toxicity at higher concentrations but also revealed chronic hepatotoxicity and underlying molecular disturbances through a 28-day exposure, thereby offering a more comprehensive mechanistic understanding of FO hazard potential. It should be emphasized that the concentrations employed in this study markedly exceed the microgram-per-liter levels commonly reported in the environment. Consequently, the observed effects should be regarded primarily as mechanistic evidence of hazard rather than as direct quantification of environmental risk, thereby furnishing essential mechanistic insight into the toxicological potential of FO. Dose–response relationships for FO toxicity in zebrafish were established at multiple levels, including embryonic lethality and malformation rates, heart rate reduction, SDH activity inhibition, oxidative stress induction, and histopathological liver damage. Transcriptomic and metabolomic analyses further dissected the underlying mechanisms by revealing perturbations in key molecular pathways and small-molecule metabolites.

**Figure 8:**
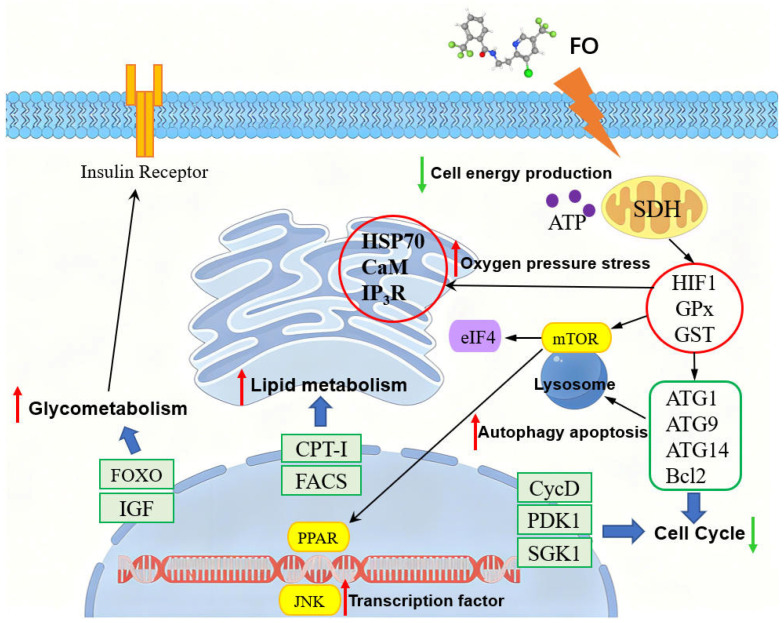
A modeling diagram of the effect of FO on hepatotoxicity in adult zebrafish. Red arrows indicate promotion and green arrows indicate inhibition.

SDHI pesticides are capable of causing developmental toxicity, immunotoxicity and even reproductive toxicity in zebrafish in the long term by altering transcription factors and the cell cycle [37,38]. The integrated transcriptomic and metabolomic analyses suggested that the long-term effects of FO on adult zebrafish involved not only disturbances in energy, amino acid, and lipid metabolism, but also disruption of cellular autophagy and transcription factor networks. As delineated in Figure 8, FO exposure directly inhibits SDH activity, leading to impaired mitochondrial ATP production and a cellular energy deficit. This energy crisis, coupled with induced oxidative stress, may trigger the up-regulation of stress-responsive (HSP70, HIF1α) and detoxification genes (GPx, GST), as supported by transcriptomic data (Figure S1) and related biological signaling pathways (Table S5). Furthermore, these molecular disturbances could be associated with the modulation of pathways governing autophagy and apoptosis. The down-regulation of autophagy-related genes (ATG1, ATG9, ATG14) and Bcl2, alongside the activation of transcription factors such as PPAR and JNK, could collectively promote lysosomal autophagy and alter cell cycle progression [39]. Concomitantly, PPAR and FOXO signaling activation may drive a metabolic reprogramming, accelerating lipid metabolism (CPT-I, FACS) and glycometabolism, potentially through the involvement of IGF and insulin receptor pathways [40]. Combined transcriptomic and metabolomic analyses outlined that FO exposure affects a variety of cellular functions starting through oxidative stress and inhibition of mitochondrial function, deeply altering zebrafish liver cell function and cell fate. This study systematically delineates the multilevel toxicity of FO in zebrafish, demonstrating that its effects are primarily driven by SDH inhibition-induced mitochondrial dysfunction and oxidative stress. Thereby, it provides essential mechanistic hazard data and lays a mechanistic foundation for understanding the potential risks associated with environmental exposure to FO.

## 5. Conclusions

This study revealed the multidimensional toxic effects of FO on zebrafish embryos and adults. FO not only significantly inhibited the SDH activity of zebrafish embryos, reducing ATP synthesis and impairing mitochondrial function, but also triggered oxidative stress, developmental malformations and behavioral abnormalities. In adult zebrafish, FO caused liver metabolic disorders, histopathological damage, and activation of cellular autophagy and PPAR, FoxO, and other signaling pathways in long-term exposure. Joint transcriptomic and metabolomic analyses showed that FO affects zebrafish growth and liver function by interfering with energy metabolism, activating detoxification systems, and altering nutrient metabolic pathways. This study is an important toxicological basis for a comprehensive assessment of the aquatic ecological risk of FO, suggesting that its environmental residues threaten aquatic organisms.

## Data Availability

The original contributions presented in this study are included in the article/Supplementary Material. Further inquiries can be directed to the corresponding author.

## Supplementary Materials

The following supporting information can be downloaded at: https://www.mdpi.com/article/10.3390/jox16020069/s1, Figure S1: Fold change of selected genes in the liver transcriptome of adult fish chronically exposed to FO.; Table S1: Acute toxicity of fluopyram to zebrafish embryos and adults; Table S2: Detection of fluopyram (FO) concentration in water exposed for 24 h; Table S3: Semi-quantitative analysis of liver histopathology in adult zebrafish after 28-day exposure to FO based on the Bernet scale; Table S4: GSEA-enriched signaling pathways in the adult fish transcriptome; Table S5: Classification of biological signaling pathways and fold-change in expression of specific genes in the liver transcriptome following chronic exposure to FO in adult fish.
