# Supplementary material for: Fluopyram Induces Multilevel Toxicity in Zebrafish: Insights from Developmental Impairment, Oxidative Stress, and Metabolic Disruption
Source: J Xenobiot. 2026 Apr 20;16(2):69. doi: 10.3390/jox16020069 (PMC13117318; doi:10.3390/jox16020069)
Supplement: Supplementary file 1 [file jox-16-00069-s001.zip › jox-4042267-supplementary.pdf]

Journal of Xenobiotics

# Supplementary Materials: Fluopyram Induces Multilevel Toxicity in Zebrafish: Insights from Developmental Impairment, Oxidative Stress, and Metabolic Disruption

**Ningbo Wang and Yingying Zhong**

**Table S1. Acute toxicity of fluopyram to zebrafish embryos and adults**

| Treatment | 96h-$LC_{10}$ (mg/L) | 96h-$LC_{50}$ (mg/L) | 95% Confidence limit (mg/L) | Regression equation | $R^2$ |
|---|---|---|---|---|---|
| Embryo | 0.47 | 3.01 | 2.67-3.35 | Y=-3.020+14.228X | 0.923 |
| Adult | 1.02 | 9.96 | 9.43-10.46 | Y=9.905+3.846X | 0.863 |

**Table S2. Detection of fluopyram (FO) concentration in water exposed for 24 hours.**

| Sample | FO concentration | Mean and standard deviation |
|---|---|---|
| FO-0.01 mg/L-0h-1 | 0.0102 | |
| FO-0.01 mg/L-0h-2 | 0.0105 | 0.0103±0.000173 |
| FO-0.01 mg/L-0h-3 | 0.0102 | |
| FO-0.01 mg/L-24h-1 | 0.0103 | |
| FO-0.01 mg/L-24h-2 | 0.0099 | 0.0099±0.000351 |
| FO-0.01 mg/L-24h-3 | 0.0096 | |
| FO-0.1 mg/L-0h-1 | 0.105 | |
| FO-0.1 mg/L-0h-2 | 0.101 | 0.1003±0.005 |
| FO-0.1 mg/L-0h-3 | 0.095 | |
| FO-0.1 mg/L-24h-1 | 0.099 | |
| FO-0.1 mg/L-24h-2 | 0.104 | 0.1±0.0036 |
| FO-0.1 mg/L-24h-3 | 0.097 | |
| FO-1 mg/L-0h-1 | 1.018 | |
| FO-1 mg/L-0h-2 | 1.067 | 1.047±0.0259 |
| FO-1 mg/L-0h-3 | 1.057 | |
| FO-1 mg/L-24h-1 | 0.979 | |

| | | |
|---|---|---|
| FO-1 mg/L-24h-2 | 0.987 | 1.0067±0.0412 |
| FO-1 mg/L-24h-3 | 1.054 | |

**Table S3. Semi-quantitative analysis of liver histopathology in adult zebrafish after 28-day exposure to FO based on the Bernet scale.**

| FO Concentration (mg/L) | Necrosis | Vacuolar Degeneration | Nuclear Pyknosis | Inflammation | Histopathological Index (Mean ± SD) |
|---|---|---|---|---|---|
| Control | 0 | 0 | 0 | 0 | 0.0 ± 0.0 |
| 0.01 | 0 | 0 | 0 | 0 | 0.0 ± 0.0 |
| 0.1 | 1 | 1 | 1 | 0 | 3.0 ± 0.8** |
| 1.0 | 2 | 2 | 3 | 2 | 9.0 ± 1.2*** |

Lesion scores: 0 (none), 1 (minimal/focal, <10%), 2 (moderate/multifocal, 10-50%), 3 (marked/diffuse, >50%). The histopathological index is the sum of all lesion scores per sample. Data are presented as mean ± standard deviation (SD) (n=5). Significance vs. control group: ** $p < 0.01$, *** $p < 0.001$ (one-way ANOVA with Tukey's post-hoc test).

**Table S4. GSEA-enriched signaling pathways in the adult fish transcriptome.**

| No | NAME |
|---|---|
| **1** | AUTOPHAGY___ANIMAL(DRE04140) |
| **2** | MTOR_SIGNALING_PATHWAY(DRE04150) |
| **3** | ADIPOCYTOKINE_SIGNALING_PATHWAY(DRE04920) |
| **4** | GLYCOSAMINOGLYCAN_DEGRADATION(DRE00531) |
| **5** | AUTOPHAGY___OTHER(DRE04136) |
| **6** | GLYCOSPHINGOLIPID_BIOSYNTHESIS___GANGLIO_SERIES(DRE00604) |
| **7** | RIG_I_LIKE_RECEPTOR_SIGNALING_PATHWAY(DRE04622) |
| **8** | FATTY_ACID_DEGRADATION(DRE00071) |
| **9** | MITOPHAGY___ANIMAL(DRE04137) |
| **10** | FATTY_ACID_METABOLISM(DRE01212) |

**Table S5. Classification of biological signaling pathways and fold-change in expression of specific genes in the liver transcriptome following chronic exposure to FO in adult fish.**

| **Biological Signaling pathway** | **Gene name** | **Fold Change** (FO vs CK_log2) |
|---|---|---|
| **Oxygen pressure stress** | HSP70 | 4.45 |
| | HIF1 | 2.03 |

| | | |
|---|---|---|
| | GPx | 1.33 |
| | GST | 1.78 |
| **Autophagy apoptosis** | mTOR | 1.88 |
| | eIF4 | 1.48 |
| | ATG1 | 1.91 |
| | ATG9 | 2.85 |
| | ATG14 | 3.52 |
| | Bcl2 | -1.63 |
| **Transcription factor** | PPARβ | 2.36 |
| | JNK | 2.03 |
| **Cell Cycle** | CycD | -1.89 |
| | PDK1 | -1.95 |
| | SGK1 | -1.17 |
| **Lipid metabolism** | CPT-I | 4.14 |
| | FACS | 2.63 |
| **Glycometabolism** | FOXO | 1.75 |
| | IGF | 1.92 |
| | INSR | 1.2 |

**Figure S1.** Fold change of selected genes in the liver transcriptome of adult fish chronically exposed to FO.

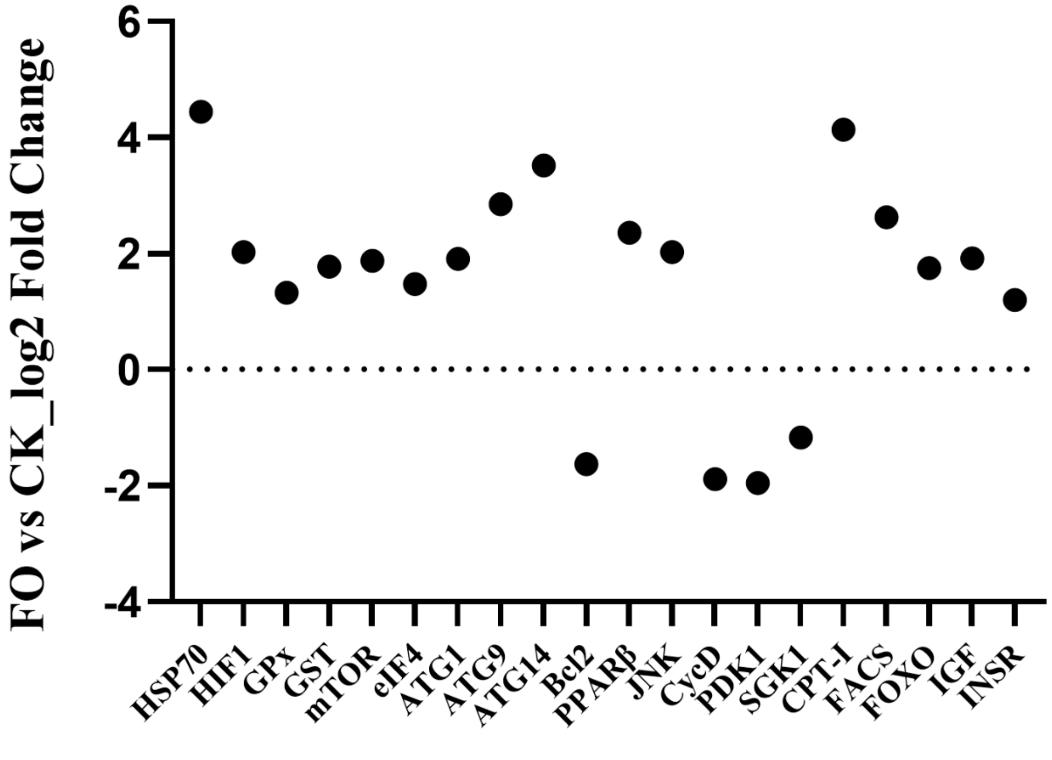


**Text S1. Acute Toxicity Testing on Zebrafish Embryos.**

The solvent control group and the exposure group in the embryo exposure experiment both used acetone as the solvent (0.0001% (v/v)). Test concentrations in the zebrafish embryonic acute toxicity assay included: control group, 1, 2, 3, 4, 5, and 6 mg/L. For heart rate statistics, exposure concentrations were set at: control group, 0.5, 1, 1.5, 2, 2.5, and 3 mg/L. Each group comprised three biological replicates (n=30). Zebrafish embryos were randomly distributed into 12-well plates, with 5 mL of zebrafish embryo medium added to each well. The medium was changed daily, and dead embryos were removed.

**Text S2. Details of the biochemical assay experiment.**

1. Superoxide Dismutase (SOD)

The activity of SOD was determined using the WST-8 method. The principle is based on the inhibition of the reduction of a water-soluble tetrazolium salt (WST-8) by superoxide anion, which is generated by the xanthine oxidase reaction. The degree of inhibition correlates with SOD activity. Briefly, the tissue supernatant was incubated with the enzyme working solution and the substrate mix. After incubation, the absorbance was measured at 450 nm.

2. Catalase (CAT)

CAT activity was assayed by measuring the decomposition of hydrogen peroxide ($H_2O_2$). The residual $H_2O_2$ after the enzymatic reaction reacts with ammonium molybdate to form a stable yellow complex. The amount of complex, measured at 405 nm, is inversely proportional to CAT activity. In brief, the sample was allowed to react with an $H_2O_2$ substrate, and the reaction was terminated by adding ammonium molybdate before absorbance reading.

3. Reduced Glutathione (GSH)

The content of GSH was quantified using the DTNB method. GSH reacts with 5,5′-dithiobis-(2-nitrobenzoic acid) (DTNB) to produce a yellow-colored 5-thio-2-nitrobenzoic acid (TNB). The intensity of the color, measured at 412 nm, is directly proportional to the GSH concentration. The procedure involved protein precipitation from the homogenate, followed by the reaction of the supernatant with DTNB reagent.

4. Succinate Dehydrogenase (SDH)

The activity of SDH was measured by a colorimetric assay. SDH catalyzes the oxidation of succinate, and the released electrons reduce an artificial electron acceptor, forming a colored formazan product. The rate of formazan generation, monitored at 600 nm, is proportional to the enzyme activity. The sample was incubated with sodium succinate and the chromogen.

5. Glucose-6-Phosphate Dehydrogenase (G6PDH)

G6PDH activity was assessed by an NADPH generation rate method. The enzyme catalyzes the oxidation of glucose-6-phosphate, simultaneously reducing $NADP^+$ to NADPH. The increase in absorbance at 340 nm due to NADPH formation is directly proportional to G6PDH activity. The assay mixture containing sample, substrate, and $NADP^+$ was prepared, and the kinetic absorbance change was recorded.

6. Alanine Aminotransferase (GPT)

GPT activity was determined using the Reitman-Frankel colorimetric method. GPT catalyzes the reaction between alanine and $\alpha$-ketoglutarate to produce pyruvate and glutamate. The generated pyruvate then reacts with 2,4-dinitrophenylhydrazine to form a brownish-red phenylhydrazone, which is measured at 505 nm. The sample was incubated with the substrate, and the reaction was stopped by adding DNPH before colorimetric measurement.

7. Aspartate Aminotransferase (GOT)

GOT activity was measured similarly by the Reitman-Frankel method. GOT catalyzes the transamination between aspartate and $\alpha$-ketoglutarate to form oxaloacetate and glutamate. The oxaloacetate is subsequently converted to a colored

product with 2,4-dinitrophenylhydrazine, and the absorbance is read at 505 nm. The procedure followed the same steps as the GPT assay with the corresponding substrate.

8. Adenosine Triphosphate (ATP)

The ATP content was determined by an enzymatic colorimetric assay. The principle involves the catalysis of ATP and creatine by creatine kinase to generate phosphocreatine. The produced phosphocreatine is then quantified using a phosphomolybdate colorimetric method, where it reacts to form a blue complex. The intensity of the color, measured at 636 nm, is proportional to the ATP concentration. Briefly, the tissue supernatant was deproteinized by heating in a boiling water bath. The resulting supernatant was incubated with substrate mixtures containing creatine kinase at 37°C. After the reaction was stopped and chromogenic agents were added, the absorbance was measured to calculate ATP content.

9. Bicinchoninic acid (BCA) assay

The total protein concentration in tissue supernatants was determined for normalization using the bicinchoninic acid (BCA) assay. Proteins reduce $Cu^{2+}$ to $Cu^{+}$ in an alkaline medium, and $Cu^{+}$ ions then chelate with BCA to form a purple-colored complex. The absorbance of this complex at 562 nm is proportional to the protein concentration. The sample was mixed with BCA working reagent, incubated, and the absorbance was measured.

**Original Images**

Figure .1D

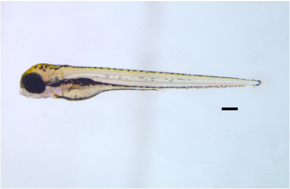

Control

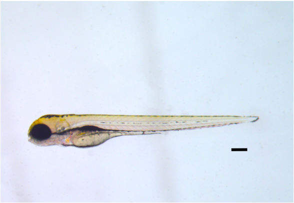

0.5 mg/L

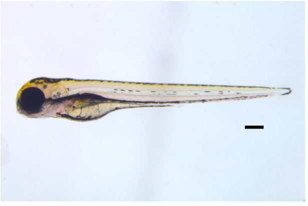

1 mg/L

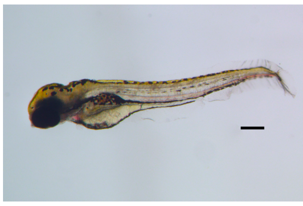

1.5 mg/L

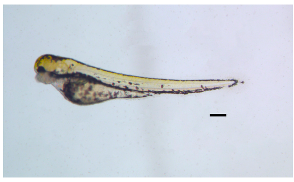

2 mg/L

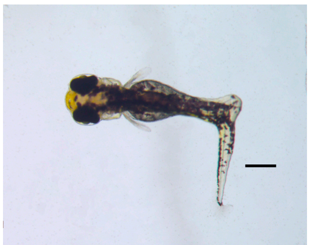

2.5 mg/L_1

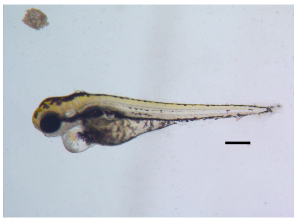

2.5 mg/L_2

**Figure .6**

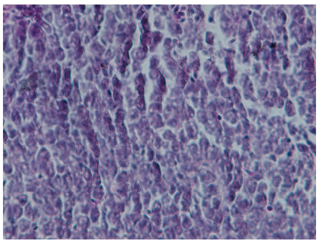

CK_1

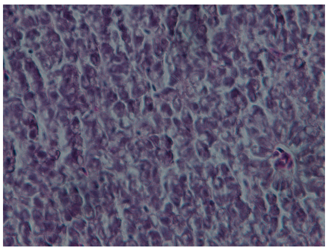

CK_2

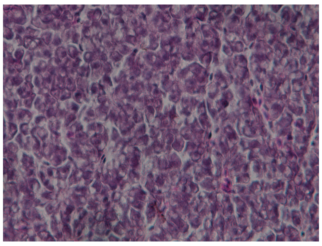

0.01 mg/L_1

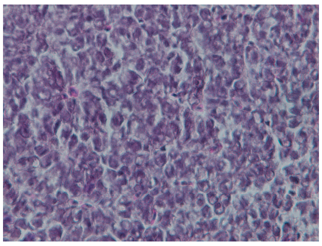

0.01 mg/L_2

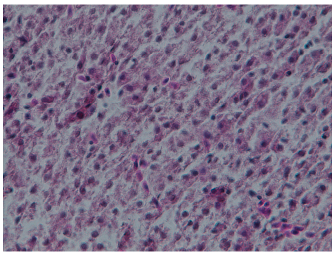

0.1 mg/L_1

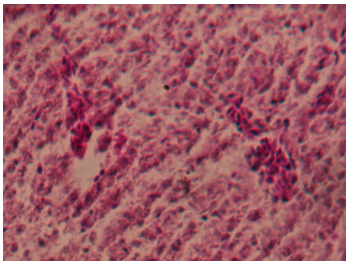

0.1 mg/L_2

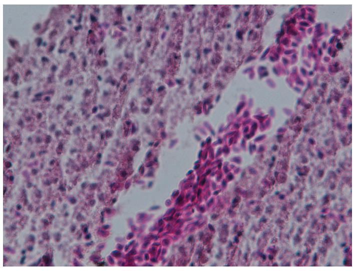

1 mg/L_1

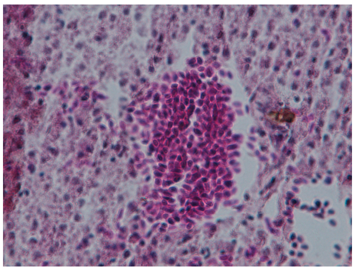

1 mg/L_2
